# Gestational Weight Gain among Healthy Pregnant Women from Asia in Comparison with Institute of Medicine (IOM) Guidelines-2009: A Systematic Review

**DOI:** 10.1155/2019/3849596

**Published:** 2019-03-03

**Authors:** Priyanka Arora, Bani Tamber Aeri

**Affiliations:** Department of Food and Nutrition, Institute of Home Economics, Delhi University, F-4, Hauz Khas Enclave, New Delhi, India

## Abstract

In 1990, Institute of Medicine (IOM) recommended gestational weight gain (GWG) ranges for women in the United States primarily to improve infant birth weight. Changes in key aspects of reproductive health of women of child bearing age, a rising prevalence of obesity, and noncommunicable diseases prompted the revision of IOM guidelines in 2009. However, there is no such recommendation available for Asian women. This systematic review assesses the utility of IOM-2009 guidelines among Indian and other Asian pregnant women in terms of maternal and fetal outcomes. 624 citations were identified using PubMed and Google Scholar, out of which 13 were included. Prospective/retrospective studies of healthy Asian women with a singleton pregnancy which specifically examined fetal-maternal outcomes relative to IOM-2009 guidelines were included.* Results*. Majority of pregnant Indian women achieved less GWG than the recommendations whereas a mixed trend was noticed among the other Asian pregnant women. The most common fetal-maternal complications among the excessive GWG women were found to be macrosomia, large for gestational age and caesarean section followed by gestational diabetes and hypertension, whereas low birth weight, small for gestational age and preterm birth, was found to be associated with low GWG women. The findings highlight the need for appropriate GWG limits across the different body mass index levels specifically for Indians and other Asian population. However, there are not enough publications regarding the utility of IOM-2009 guidelines among the Indian and other Asian women. Thus, higher-quality researches are warranted in future to further validate the findings of the present review.

## 1. Introduction

Nutritional status of women is of much importance for the well-being of both the mother and the developing fetus. Two independent factors—prepregnancy body mass index (BMI) and weight gain during pregnancy—play important roles in determining the pregnancy outcome [1]. According to National Family Health Survey-4 (NFHS, 2015-16), 22.9% of women in childbearing age in India are underweight (BMI <18.5kg/m^2^), whereas a rise has been observed from 12.6% (NFHS-3, 2005-06) to 20.7% among overweight/obese (BMI-≥25kg/m^2^) women (NFHS-4) [2, 3]. Prepregnancy underweight (UW) has been shown to increase the risk of preterm birth and and low birth weight (LBW) [4] whereas prepregnancy overweight/obesity is a risk factor for gestational diabetes mellitus (GDM), gestational hypertension (GHTN), and preeclampsia [5, 6]. Gestational weight gain (GWG) results from various structural and functional modifications that occur in a woman's body to meet the nutritional requirements of pregnancy including fetal and placental growth, increase in amniotic fluid, placenta, increased blood volume, increased adipose tissue, uterine and mammary growth, etc. [7]. Using this knowledge, recommendations for GWG have been developed [8]. Gaining desirable GWG is considered to be effective in supporting the growth and development of the fetus and it may also influence the body composition in childhood and later life [9–11]. Studies have also shown that excessive GWG has been associated with a higher fat mass in childhood and greater BMI and fat mass in later adulthood [12, 13]. The rate of weight gain varies throughout pregnancy and its timing during pregnancy also has an impact on birth weight [14].

During the 20^th^ century, recommendations for maternal weight gain during pregnancy were controversial, ranging from rigid restriction to encouragement of ample gain. In 1990, the Institute of Medicine (IOM) recommended GWG ranges with the primary goal of improving infant birth weight. Though the IOM guidelines were widely adopted, these were not universally accepted [27, 28]. Later on, gradual change in key aspects of the reproductive health of women of childbearing age especially increases in advanced maternal age; a rising prevalence of obesity, diabetes mellitus, hypertension, and other chronic noncommunicable diseases prompted the revision of the IOM guidelines in 2009. These evidence-based recommendations were designed to help the maternity care providers to assist their patients in managing pregnancy-associated weight [10]. The guidelines offer specific weekly (kg/wk) and absolute (total kg) weight gain recommendations based on a woman's pregravid BMI [29] and also provide a specific range of weight gain for overweight and obese women that were previously lacking [30]. In order to provide consistency in women's care, these new IOM- 2009 guidelines are based on the BMI cut-offs developed by the World Health Organisation [31] and adopted by the National Heart, Lung, and Blood Institute [32] that are widely used in the United States and elsewhere which is described as below: <18.5 kg/m^2^ (underweight), 18.5–24.9 kg/m^2^ (normal weight), 25–29.9 kg/m^2^ (overweight), and ≥30 kg/m^2^ (obese) [10]. According to these guidelines, the range of weight gain recommended for underweight (UW) is 12.5 -18 kg, normal weight (NW) women is 11.5-16 kg, and overweight women (OW) is 7-11.5 kg whereas the obese (OB) women are recommended to achieve only 5-9 kg of weight throughout pregnancy. The IOM-2009 are the most widely accepted recommendations for GWG [33], but it is not clear if these guidelines are also applicable to developing countries [34]. This is mainly because the BMI classification for Asians [35] is different from WHO- BMI cut-offs recommended for the West. The weight gain recommendations by the IOM are in turn, based on Western WHO BMI cut-offs, making it difficult to compare, translate, or generalize their findings to Asian Indians [16]. In addition, there is also no such recommendation for GWG cut-off points available for all Asians [19]. The present review focuses on pregnancy outcome and other pregnancy-related complications for singleton pregnancies among Indian and other Asian population with respect to IOM-2009 weight gain recommendations. It assesses the utility of IOM guidelines-2009 among the pregnant women of India and other Asian countries in terms of maternal and fetal outcomes. While other systematic reviews have assessed GWG with respect to IOM-1990 guidelines, to our knowledge, this is the first review exploring relationships between prepregnancy BMI and IOM-2009 GWG limits in Indian settings. Therefore, purpose of this review was to compare GWG among healthy pregnant women across different BMI and compare it with the IOM guidelines-2009. An effort was also made to evaluate associate feto-maternal outcomes with GWG above, within and below the IOM- 2009 guidelines. This review is reported in concordance with the Preferred Reporting Items for Systematic Reviews and Meta-Analyses statement (PRISMA) [36].

## 2. Materials and Methods

### 2.1. Eligibility Criteria

The protocol of selection criteria was developed on the basis of population, intervention, comparators, and outcomes (PICO) questions. Any retrospective or prospective study which recruited (*POPULATION*) healthy pregnant women with a singleton pregnancy and specifically examined (*OUTCOME*) foetal-maternal outcomes and any other pregnancy-related complications (GDM, GHTN, preeclampsia/eclampsia, caesarean section, and thyroid dysfunction) relative to the IOM's recommended weight gain ranges-2009 on the basis of (*COMPARISON*) different prepregnancy BMI levels were eligible for the inclusion in systematic review. According to IOM-2009 [10], teenagers who are pregnant are recommended to use the adult BMI categories to determine their weight gain range until more research is done to determine whether special categories are needed for them. However, the studies that were restricted to adolescents and in which the mean age of the total population was <18 years were also excluded as to date there are no recommendations available exclusively for teenage pregnancy. The studies comprised of mothers who were pregnant with twins or multiple pregnancies were not taken into the consideration. In addition, (*INTERVENTION)* RCT in which the impact of nutrition education/lifestyle intervention/supplementation was assessed on pregnancy outcome or pregnancy-related complication were also included. The studies that were not related to maternal weight gain or focused on the countries other than Asia were excluded. Further, no systematic review or meta-analysis was considered for analysis. The articles published only in the English language were taken into consideration. The PubMed library database and Google Scholar were searched using combinations of the following text and Mesh terms: “Gestational weight gain,” “Weight gain during pregnancy,” “Maternal weight gain”, “IOM Guidelines”, “Asian”, and “India”. The articles published during January 2010-March 2018 were taken into consideration and the final search was done on 10th March 2018. This time frame was selected according to the release of the new 2009 IOM guidelines.

### 2.2. Study Selection and Data Collection

624 studies were screened by the authors. Following the screening of titles and abstracts, the full text was read in order to identify the articles which fulfilled the inclusion criteria for the final analysis. However, 18 articles were selected based upon the eligibility criteria and only 13 articles were selected for the final review. The study details (publication year, study design, sample size, subject s' characteristics, and outcomes) were aggregated in Microsoft Office Excel 2010 for summary and analyses. Measures of central tendency such as mean, median, range, and percentage were used to represent and summarise the data. A summary table was designed to gather the study characteristics of interest (Table 1)

### 2.3. Data Extraction and Quality Assessment

Information was documented by the reviewers from various studies using a piloted data extraction form. Beside this, the quality of observational studies (cohort/cross-sectional studies) was assessed by using Newcastle-Ottawa Scale (NOS) [37] whereas the quality of RCTs was assessed using the Cochrane Risk of Bias tool (CRBT) [38]. A NOS score was computed on the basis of selection, comparability, and outcome criteria. Studies with a score of 7 or more were considered to be “good” [39]. The RCTs were categorized as poor, fair, or good quality using CRBT on the basis of selection bias, performance bias, detection bias, attrition bias, and reporting bias [38].

### 2.4. Risk Bias across Studies

There was a difference in the BMI reference used in Indian and other Asian studies to categorize the pregnant women. However, to overcome this problem, cut-off criteria chosen for defining weight category have been used for understanding the impact of GWG on pregnancy outcome.

## 3. Results

### 3.1. Study Selection

**Figure 1:**
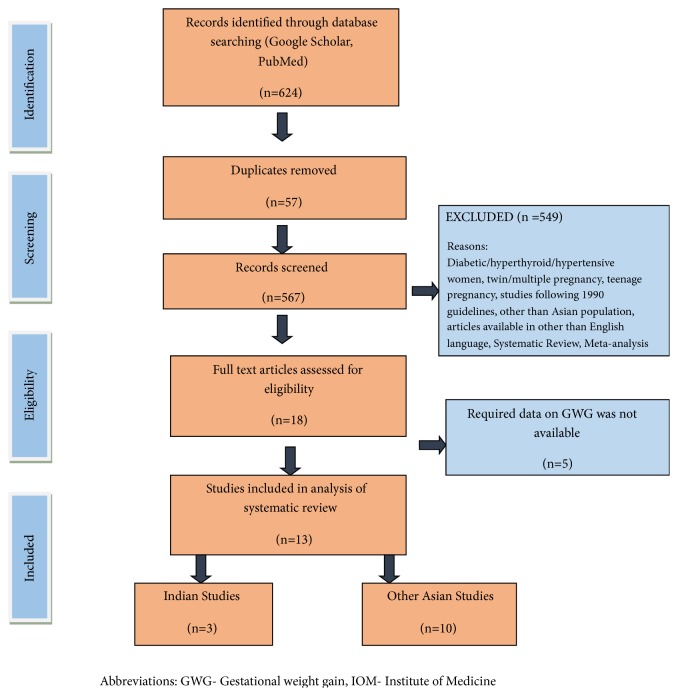
Flow diagram of study selection process.

The flow diagram (Figure 1) outlines the process of identification and selection of studies. The search yielded 624 citations, out of which 611 were excluded for the reasons shown in Figure 1. Accordingly, 13 studies met the inclusion criteria and were included in the systematic review.

### 3.2. Study Characteristics

Among 13 selected articles, 3 were from India [16, 21, 22], and remaining 10 studies were from other Asian countries [14, 15, 17–20, 23–26]. There were 10 cohorts [14, 16, 17, 19–25], 2 randomised control trials (RCTs) [15, 26], and only 1 cross-sectional study [18]. One of the RCTs [26] adopted a retrospective design which used the secondary data obtained from an RCT, PRECONCEPT study, evaluating the effects of preconception micronutrient supplementation on maternal and child health outcomes [40]. Total 2,76,107 pregnant women (mean 21239, median 1436) participated with an age range of 18-44 years in all 13 included studies. Indian studies are comprised of least participants (mean 1017, 200 median) as compared to other Asian studies (mean 27306, median 3085).

There was a difference in the gestational age at which enrolment was carried out in the studies. For instance, 3 studies recruited the women within the first 12 weeks of pregnancy [15, 19, 20], whereas in 2 studies, women within the first 10 weeks [22] and ≥28 weeks of pregnancy [25] were enrolled for recruitment. Another study administered the validated protocol containing sociodemography and anthropometry measurements only among the pregnant women who belong to the 3^rd^ trimester [21]. In contrary to this, a study [26] recruited nonpregnant women who were planning to have children in the upcoming year and were followed up to 3 months postpartum. A retrospective investigation is comprised of the records provided by the Pregnancy Birth Registry System, which collected information on successive deliveries occurring at gestational week 22 or later [17]. In other retrospective studies interviews were conducted and maternal records were used to retrieve the relevant information from the women shortly after the delivery [14, 18, 23–25].

In a majority of studies, maternal-related information and data regarding the pregnancy outcome were obtained from the past medical records [16, 17, 19, 20, 25]. In another study, interviews were conducted at the time of enrolment (32-34 weeks) of pregnant women to obtain the information about their demographic profile, history of past illness, anthropometric measurements whereas BMI and details regarding the biochemical parameters of the first trimester were retrieved from their antenatal cards [21]. Similarly, in other few studies, the relevant details of the participants were retrieved from the past medical records whereas information related to prepregnancy weight and height was self-reported either through a telephonic interview [23] or a face to face interview [24]. In another study also prepregnancy weight was self-reported but interviews were held during pregnancy to collect the maternal information and other details about fetal-maternal outcomes were obtained shortly after the delivery [15, 18, 26]. Beside this, it was observed in another study [22] that during the first visit in early pregnancy their height and weight were measured and BMI was calculated and information related to pregnancy outcome was gathered during the postpartum phase. But there is no information available in a study [14] regarding the source of data on maternal height and weight.

The quality score of observational studies ranged from 3-8 (Table 1). 8 out of 11 studies [14, 16–18, 21–23, 25] received a quality score of <7 which indicated their poor quality in comparison to remaining 3 [19, 20, 24] studies which scored ≥7, whereas the quality of two RCTs was found to be fair [15] and good [26], respectively. Thus, the included studies had, on average, a low quality. Majority of studies did not receive any quality points for not providing the details about the number of participants lost due to follow-up, representativeness of the community/population from where the sample has been retrieved and blinding of participants and other personnel.

### 3.3. Risk Bias within Studies

One Indian study [16] followed WHO Asia Pacific BMI cut points [35] and classified pregnant women into four categories but the cut-offs were different- <18.5 kg/m^2^, 18.5-22.9kg/m^2^, 23-24.9kg/m^2^, and ≥25kg/m^2^. The other 2 Indian studies [21, 22] and 6 Asian studies [14, 15, 17–19, 23] classified the pregnant women BMI into 4 categories as per WHO- <18.5kg/m^2^, 18.5-24.9 kg/m^2^, 25-29.9 kg/m^2^, and ≥30kg/m^2^. Beside this, other 3 Asian cohort studies [20, 24, 25] divided Chinese women into four groups based on according to BMI categories defined by the Working Group on Obesity in China [41]- < 18.5 kg/m^2^, 18.5 kg/m^2^ ≤ BMI < 24.0 kg/m^2^, 24.0 kg/m^2^ ≤ BMI < 28.0 kg/m^2^, and ≥ 28.0 kg/m^2^. Another Asian, RCT study [26] conducted in Vietnam used different BMI levels - <18.5 kg/m^2^, 18.5-23 kg/m^2^, and > 23 kg/m^2^.

### 3.4. Adequacy of GWG among Asian Pregnant Women according to IOM, 2009 Guidelines

A retrospective study [17] conducted among the Japanese pregnant women who were included in the Japan Society of Obstetrics and gynecology registry system illustrated that majority of UW (76.3%), NW (63.9%), OW (39.8%), and OB women (46.3%) gained less weight than the IOM recommendations. After analyzing the retrospective records [16] of 2728 pregnant women attending antenatal clinics in Chennai, India, it was seen that, apart from OB women, the majority of UW, NW, and OW women achieved less GWG than the IOM recommendations. In another study [14] nearly half (46.5%) of the women had a normal prepregnancy BMI but majority of them (79.2%) also gained less than the NW gain prescribed. Similar results were obtained in other studies [22, 26] as well which showed that majority of women (73.4%) and (59%) were found to achieve less than the GWG recommendations. Another longitudinal study [21], carried out on 124 booked antenatal cases at a tertiary care center, illustrated that 55.3% women gained gestational weight <8.9kg, 36.6% women gained 9-14.9 kg, and only 10% gained >15kg. It also indicated that more than half of them had a less than prescribed weight gain.

On the contrary, studies conducted among the Chinese population revealed that majority of pregnant women gained above the IOM recommendations [19, 20, 25]. A similar trend was seen in another previous study conducted among live singleton pregnant women at Malaysia which showed that majority of NW (38.9%), OW (56%), and OB (52.9%) gained more than optimal GWG [23].

There are few studies which concluded that majority of the women were able to meet the IOM recommendations. This can be evident by the findings collected from the Chinese population [24], which illustrated that majority of pregnant women (43.5%) were able to have adequate GWG. Similarly, another study [18] conducted among the population residing in Korea showed that the women who gained below the recommended weight gain during pregnancy, within, and over were 25.3%, 38.7%, and 36.0%, respectively. In a RCT conducted among 90 pregnant women, proportion of women within the IOM recommendations were higher in intervention group (51.1%) which received individualized lifestyle intervention focusing on healthy lifestyle, diet, and exercise along with the weight monitoring during 12-15,16-18,20-24, and 37 weeks of gestation than control group (28.9%) which only received routine antenatal care [15].

### 3.5. Feto-Maternal Outcomes Associated with GWG

Data collected from the mother-infant pairs demonstrated that women with excessive GWG exhibited an increased risk of macrosomia and LGA infants whereas women with inadequate GWG exhibited increased risks of SGA infants when compared to women who had adequate GWG [24]. In another study [22], the incidence of GDM was higher (26.1%) among excessive GWG than low GWG (13.6%) and incidence of GHTN was higher (21.7%) in excessive GWG than adequate weight gain women (6.8%). Besides this, a similar trend was observed in other two studies [17, 22] which found that incidence of preterm birth, LBW, and SGA increased when GWG was lower than optimum GWG. In another study, it was observed that likelihood of birth of SGA infant was 2.5 times higher for women who gained below the IOM recommended guidelines in comparison to women who gained within the optimal limits [26]. Similarly in another cohort conducted among the Pakistan women population exhibited that the women not reaching the optimal weight as per IOM were at a greater risk of preterm delivery in comparison to those gaining within the recommended ranges (17.8 versus 15.0%) and LBW (8.7% versus 7.3%). In contrast, women who gained weight above recommendation were more likely to have LGA (10.7% versus 5.8%) than women with recommended weight gain [14]. Thus, not only excessive weight gain but inadequate GWG may also lead to the poor fetal-maternal outcome.

On correlating prepregnancy BMI, GWG, and fetal-maternal outcome, a higher risk of preterm delivery, CS, macrosomia, and preeclampsia for OB women who gained more weight was observed [16]. Similarly, after analyzing the healthcare records of 33,973 pregnant women it was well demonstrated that women with both prepregnancy obesity and excessive GWG had 2.2-5.9-fold higher risk of GDM, CS, LGA, GHTN, and macrosomia compared with NW women and adequate GWG [20]. The data collected from another retrospective study conducted among 436 Malaysian women found a higher frequency of macrosomia among the overweight women who had gained excessive GWG and further found a higher frequency of LBW among NW women who had low GWG. In addition to this, the majority of the normal weight, overweight, and obese women who had gained excessive GWG had undergone CS rather than normal delivery [23]. In another study, it was observed that, in all the prepregnancy BMI category groups, excessive GWG was associated with higher frequency of LGA and macrosomia whereas poor weight gain correlated with SGA and preterm and optimal weight gain within the recommended range was found to be associated with better outcome [17].

### 3.6. Risk of Bias across Studies

Among 13 included studies, 2 studies [16, 26] used different BMI levels whereas 8 studies [14, 15, 17–19, 21–23] used same BMI levels to categorized the pregnant women. The remaining three studies [20, 24, 25] conducted among the Chinese population divided women based on BMI categories defined by the Working Group on Obesity in China [41]. Overall, Indian studies had enrolled less pregnant women (median 200) than other Asian studies (median 3085). BMI classification across the studies is not standardized leaving difficulty in obtaining the inference from the studies.

## 4. Discussion

### 4.1. Summary of Evidence

The initial guidelines by the IOM in 1930 recommended that pregnant women should gain 6.8 kg irrespective of weight status [31]. With increasing prevalence of obesity and an increasing trend in the birth of macrosomic infants, these guidelines were revised in 1990 and 2009 [10, 32]. This systematic review was aimed at assessing the utility of the IOM guidelines-2009 and explores the relationship between adequate /inadequate GWG and pregnancy outcome among the pregnant women of India and other Asian countries. The study may significantly contribute to laying a foundation required to refine current guidelines available for pregnant women for a better outcome, especially in Asian countries.

The key finding was that majority of Indian pregnant women achieved less GWG than the recommendations whereas a mixed trend was noticed among the other Asian pregnant women. It was found that women who gained excessive or inadequate gestational weight than the IOM, 2009 recommendations were associated with poor pregnancy outcome compared to the women who had gained weight adequately. Similar findings were observed in a recent systematic review and meta-analysis conducted from diverse international cohorts which had demonstrated a greater risk of adverse maternal and infant outcome among the women who gained either below or above the guidelines, though higher proportion (47%) had gained above and lesser (23%) had gained below the guidelines [42]. It was observed that 9%–70% of OW/OB women tend to gain excessive weight than the IOM-2009 limits (Table 2). This further exposes them to risks of adverse pregnancy outcome. Therefore, OW/OB women who gained more weight than recommended were found to be at a high risk of developing adverse pregnancy outcomes which includes macrosomia [30, 32, 34, 37] GDM, PIH, CS [34], pre-term labor and pre-eclampsia [30]. In an earlier systematic review also, which observed GWG above guidelines, was found to be associated with increased LGA risk [42]. Among the UW and NW women, the incidences of LGA and macrosomia were significantly higher with increasing GWG and the incidences of SGA, preterm, and LBW were higher in the group with GWG below the optimal range. Though a major proportion of NW and OW women gained less weight than recommended, the less weight gain was associated with less risk for CS and macrosomia [16] whereas in another study conducted in past [17], it was found to be associated with higher risk of CS, GDM, and SGA.

Irrespective of BMI, the most common fetal-maternal complications among the excessive GWG women were found to be macrosomia, LGA, and CS followed by GDM and GHTN whereas, among low GWG women, LBW, SGA, and preterm birth were found to be more common. The same trend was noticed in a prior systematic review which indicated 5% higher risk of both SGA and preterm birth among the low GWG women whereas weight gain above guidelines was associated with 4%, 6%, and 4% higher risk of LGA, macrosomia, and CS, respectively [42].

In addition to this, the lifestyle intervention was found to be successful in improving the lifestyle behaviour during pregnancy and it also increased the appropriate GWG for prepregnancy BMI. Thus the lifestyle intervention offered within the scope of antenatal care was found to be effective in terms of ensuring optimal GWG and developing a healthy lifestyle [15]. The WHO also has prioritized achievement of ideal BMI prior to conception and prevention of excess GWG [43]. Therefore, health care provided by the medical professionals can help pregnant women to achieve the optimum weight gain that may reduce the likelihood of poor pregnancy outcome after following the appropriate GWG recommendations if available for that specific population.

### 4.2. Strength and Limitation

Strengths of this review are selecting two comprehensive databases which have been used for searching scientifically sound research studies. The number of articles chosen had high quality. Further the data used was based upon current IOM-2009 guidelines and studies which were strictly adherent were obtained. The quality of studies was assessed using 2 scientific tools, namely, NOS and CRBT which helped in ensuring high quality of systematic review. The current review clearly shows that there is difference in BMI cut-off among the population which represents major limitation for study. Another limitation is that meta-analysis of the findings was not performed due to heterogeneity in the studies which were included (e.g., different BMI levels). All these studies include women mostly from an urban area who have good healthcare access, education level, and better financial condition. This excludes a major proportion of pregnant women especially in rural parts of India. This further necessitates studies on the heterogeneous population with different financial status and education level.

### 4.3. Conclusion

According to the trend of GWG seen among the Indian and Asian women, it was found that weight gain lower than the IOM-2009 recommendations or more than the recommendation could lead to poor pregnancy outcome. This highlights the need for appropriate GWG limits across the different BMI levels specifically for Indians and other Asian population. Dietary counseling must be followed throughout the conception along with the adequate physical activity required by the women to achieve the recommended adequate GWG in order to avoid the adverse fetal-maternal outcomes. However, there are not enough publications regarding the utility of IOM-2009 guidelines among the Indian and other Asian women. Thus, higher-quality researches are warranted in future to further validate the findings of the present review.

## Figures and Tables

**Table 1:** Study characteristics of included studies^a^.

Reference	Study design	n	Age (years)	Mean GWG (kg)	Quality Rating
[15]	Prospective RCT	102	Control group: 24.28 (NR) Intervention group: 24.31(NR)	Control group-12.29 Intervention group:12.45	Fair

[16]	Retrospective Cohort	2,728	27.4 (NR)	UW- 9.3, NW- 9.1, OW- 9.0 OB- 8.5	4

[17]	Retrospective Cohort	97,157	31.8 (NR)	UW-10.27, NW- 10.11, OW- 7.98 OB- 5.5	4

[18]	Retrospective Cross-sectional	75	NR (25-40)^b^	Unemployed women^e^- 14.2 Employed women- 14.3	3

[19]	Retrospective Cohort	48,867	NR (NR)	UW- 15.47, NW- 14.51, OW- 12.95 OB- 12.69	7

[20]	Retrospective Cohort	33,973	27.6 (NR)	UW- 16.5, NW- 17.7, OW- 18.1 OB- 17.3	7

[21]	Prospective Cohort	123	25.09 (NR)^b^	9.73^d^	6

[14]	Retrospective Cohort	4,735	28.2 (19-35)^c^	8.5^d^	5

[22]	Prospective Cohort	200	NR (NR)	NR	4

[23]	Retrospective Cohort	436	NR (NR)^b^	NR	3

[24]	Retrospective Cohort	510	NR (18-44)	15.1^d^	8

[25]	Retrospective Cohort	85,765	NR (NR)	UW- 19, NW- 17.2, OW- 14.4 OB- 13.5	4

[26]	Retrospective RCT	1,436	25.8 (NR)	10^d^	good

GWG: gestational weight gain, NR: not reported, NW; normal weight, OB; obese, OW: overweight, RCT: randomised control trial, UW: underweight.

All values are mean; ranges in parenthesis (unless otherwise indicated).

^a^All the participants were healthy Asian women with a singleton pregnancy.

^b^Age was reported stratified by exposure or outcome.

^c^In study, vast majority of the subjects (90.7%) were between 19 and 35 years.

^d^In study, mean GWG was reported but it was not stratified by different levels of body mass index (BMI).

^e^In study, mean GWG was reported stratified by employment status of participants.

**Table 2:** Comparison of gestational weight gain (GWG) based on body mass index (BMI).

Author/Year/Number of women included	Inadequate weight gain	Adequate weight gain	Excessive weight gain
	N (%)	N (%)	N (%)
Enomoto, 2016 [17], n= 97,157	UW 13529 (76.3)	UW 3783 (21.3)	UW 412 (2.3)
	NW 44189(63.9)	NW 20835 (30.1)	NW 4102 (5.9)
	OW 2990 (39.9)	OW 2810 (37.5)	OW 1702 (22.7)
	OB 1297 (46.2)	OB 853 (30.4)	OB 655 (23.4)

Bhavadharini, 2017 [16], n=2,728	UW 102 (66.2)	UW 47 (30.5)	UW 5 (3.3)
	NW 550(69.5)	NW 185 (23.4)	NW 56 (7.1)
	OW 347 (68.8)	OW 113(22.5)	OW 44(8.7)
	OB 440(34.4)	OB 474(37.1)	OB 365(28.5)

Munim, 2012, [14], n=4735	UW 112 (78.0)	UW 25 (17.4)	UW 7 (4.6)
	NW 1740 (79.2)	NW 359 (16.3)	NW 104 (4.5)
	OW 483 (35.9)	OW 647 (48.2)	OW 214 (15.9)
	OB 147 (14.1)	OB 585 (56.0)	OB 312 (29.9)

Pal, 2017, [22], n= 200	UW 12 (92.3)	UW 1 (7.7)	UW 0 (0)
	NW 79 (75.2)	NW 22 (21)	NW 4 (3.8)
	OW 25 (38.5)	OW 29 (44.6)	OW 11 (16.9)
	OB 2 (11.8)	OB 7 (41.2)	OB 8 (47.1)

Rozlan, 2012, [23], n=436	UW 41 (62.1)	UW 13 (19.7)	UW 12 (18.2)
	NW 53 (30.3)	NW 54 (30.9)	NW 68 (38.9)
	OW 22 (17.6)	OW 33 (26.4)	OW 70 (56)
	OB 12 (17.1)	OB 21 (30)	OB 37 (52.9)

Li. C, 2015, [19], n=48,867	UW 1728 (26.90)	UW 2859 (44.50)	UW 1837(28.60)
	NW 9975 (26.70)	NW 13599 (36.40)	NW 13785(36.90)
	OW 495 (11.00)	OW 1381 (30.70)	OW 2621(58.30)
	OB 8 (1.40)	OB 161 (27.40)	OB 418 (71.20)

NW: normal weight, OB: obese, OW: overweight, and UW: underweight.

## References

[1] Choi S., Park I., Shin J. (2011). The effects of pre-pregnancy body mass index and gestational weight gain on perinatal outcomes in Korean women: a retrospective cohort study. *Reproductive Biology and Endocrinology*.

[2] International Institute for Population Sciences (IIPS) (2015-2016). *National Family Health Survey (NFHS-4)*.

[3] International Institute for Population Sciences (IIPS) (2007). *National Family Health Survey (NFHS-3), 2005. 06: India: Volume I*.

[4] Han Z., Mulla S., Beyene J., Liao G., McDonald S. D., Knowledge Synthesis Group (2011). Maternal underweight and the risk of preterm birth and low birth weight: A systematic review and meta-analyses. *International Journal of Epidemiology*.

[5] Doherty D. A., Magann E. F., Francis J., Morrison J. C., Newnham J. P. (2006). Pre-pregnancy body mass index and pregnancy outcomes. *International Journal of Gynecology and Obstetrics*.

[6] Hoff G. L., Cai J., Okah F. A., Dew P. C. (2009). Pre-pregnancy overweight status between successive pregnancies and pregnancy outcomes. *Journal of Women's Health*.

[7] Waring M. E., Moore Simas T. A., Liao X. (2013). Gestational weight gain within recommended ranges in consecutive pregnancies: A retrospective cohort study. *Midwifery*.

[8] Godoy A., Nascimento S., Surita F. (2015). A systematic review and meta-analysis of gestational weight gain recommendations and related outcomes in Brazil. *Clinics*.

[9] Olafsdottir A. S., Skuladottir G. V., Thorsdottir I., Hauksson A., Steingrimsdottir L. (2006). Combined effects of maternal smoking status and dietary intake related to weight gain and birth size parameters. *British Journal of Obstetrics and Gynaecology*.

[10] Rasmussen K. M., Yaktine A. L., Institute of Medicine Committee to reexamine iom pregnancy weight guidelines. *Food and Nutrition Board and Board on Children, Youth, and Families*.

[11] Drehmer M., Camey S., Schmidt M. I. (2010). Socioeconomic, demographic and nutritional factors associated with maternal weight gain in general practices in Southern Brazil. *Cadernos de Saúde Pública*.

[12] Reynolds R. M., Osmond C., Phillips D. I., Godfrey K. M. (2010). Maternal BMI, parity, and pregnancy weight gain: influences on offspring adiposity in young adulthood. *The Journal of Clinical Endocrinology & Metabolism*.

[13] Crozier S. R., Inskip H. M., Godfrey K. M. (2010). Weight gain in pregnancy and childhood body composition: findings from the southampton women’s survey. *American Journal of Clinical Nutrition*.

[14] Munim S., Maheen H. (2012). Association of gestational weight gain and pre-pregnancy body mass index with adverse pregnancy outcome. *Journal of the College of Physicians and Surgeons--Pakistan: JCPSP*.

[15] Aşcı Ö., Rathfisch G. (2016). Effect of lifestyle interventions of pregnant women on their dietary habits, lifestyle behaviors, and weight gain: a randomized controlled trial. *Journal of Health, Population and Nutrition*.

[16] Bhavadharini B., Anjana R., Deepa M. (2017). Gestational weight gain and pregnancy outcomes in relation to body mass index in Asian Indian women. *Indian Journal of Endocrinology and Metabolism*.

[17] Enomoto K., Aoki S., Toma R. (2016). Pregnancy outcomes based on pre-pregnancy body mass index in Japanese women. *PLoS ONE*.

[18] Jung Y., Choi M. (2017). Nutrient intake according to weight gain during pregnancy, job status, and household income. *Clinical Nutrition Research*.

[19] Li C., Liu Y., Zhang W., Obukhov A. G. (2015). Joint and independent associations of gestational weight gain and pre-pregnancy body mass Index with outcomes of pregnancy in chinese women: a retrospective cohort study. *PLoS ONE*.

[20] Li N., Liu E., Guo J. (2013). Maternal prepregnancy body mass index and gestational weight gain on pregnancy outcomes. *PLoS ONE*.

[21] Misra A., Ray S., Patrikar S. (2015). A longitudinal study to determine association of various maternal factors with neonatal birth weight at a tertiary care hospital. *Medical Journal Armed Forces India*.

[22] Pal R., Maiti M., Roychoudhury B., Sanyal P., Chowdhury B. (2017). Association of Pregestational BMI and Antenatal Weight Gain With Pregnancy Outcome: A Prospective Observational Cohort Study. *International Journal of Women's Health and Reproduction Sciences*.

[23] Rozlan N., Mohd Abd M H. A., Abas S. S., Danis A., Anuar Md. K. (2012). The association of gestational weight gain and the effect on pregnancy outcome defined by BMI group among women delivered in hospital Kuala Lumpur (HKL), Malaysia: A retrospective study. *Asian Journal of Clinical Nutrition*.

[24] Xiao L., Ding G., Vinturache A. (2017). Associations of maternal pre-pregnancy body mass index and gestational weight gain with birth outcomes in Shanghai, China. *Scientific Reports*.

[25] Yang S., Peng A., Wei S. (2015). Pre-pregnancy body mass index, gestational weight gain, and birth weight: a cohort study in China. *PLoS ONE*.

[26] Young M. F., Hong Nguyen P., Addo O. Y. (2017). Timing of gestational weight gain on fetal growth and infant size at birth in Vietnam. *PLoS ONE*.

[27] Abrams B., Altman S. L., Pickett K. E. (2000). Pregnancy weight gain: still controversial. *American Journal of Clinical Nutrition*.

[28] Institute of Medicine (pp. 1-233, 1990). *National Academy of Sciences, Subcommittee on Nutritional Status and Weight Gain during Pregnancy*.

[29] Ferraro Z. M., Contador F., Tawfiq A., Adamo K. B., Gaudet L. (2015). Gestational weight gain and medical outcomes of pregnancy. *Obstetric Medicine*.

[30] Radhakrishnan U., Kolar G., Nirmalan P. K. (2014). Cross-sectional study of gestational weight gain and perinatal outcomes in pregnant women at a tertiary care center in southern India. *Journal of Obstetrics and Gynaecology Research*.

[31] WHO Physical Status The use and interpretation of anthropometry. *Report of a WHO Expert Committee*.

[32] National Heart Lung (1998). Obesity education initiative expert panel on the identification, evaluation, and treatment of overweight and obesity in adults. *Clinical Guidelines on the Identification, Evaluation, and Treatment of Overweight and Obesity in Adults*.

[33] Davies G. A., Maxwell C., McLeod L. (2010). SOGC Clinical Practice Guidelines: Obesity in pregnancy. *International Journal of Gynecology & Obstetrics*.

[34] Wen T., Lv Y. (2015). Inadequate gestational weight gain and adverse pregnancy outcomes among normal weight women in China. *International Journal of Clinical and Experimental Medicine*.

[35] Bassett J., International Diabetes Institute, World Health Organization Regional Office for the Western Pacific, International Association for the Study of Obesity, International Obesity Task Force The Asia-Pacific perspective: redefining obesity and its treatment.

[36] Moher D., Liberati A., Tetzlaff J., Altman D. G. (2009). Preferred reporting items for systematic reviews and meta-analyses: the PRISMA statement. *PLoS Medicine*.

[37] Wells G. A., Shea B., O'Connell D., Peterson J., Welch V., Losos M. The Newcastle-Ottawa Scale (NOS) for assessing the quality if nonrandomized studies in meta-analyses. http://www.ohri.ca/programs/clinical_epidemiology/oxford.htm.

[38] Shuster J. J., Higgins J. P. T., Green S. (PP. 126-130, 2011). *Review: Cochrane Handbook for Systematic Reviews for Interventions*.

[39] McPheeters M. L., Kripalani S., Peterson N. B. (2012). Quality improvement interventions to address health disparities. Closing the quality gap: revisiting the state of the science. *Evidence Report*.

[40] Nguyen P. H., Lowe A. E., Martorell R. (2012). Rationale, design, methodology and sample characteristics for the Vietnam pre-conceptual micronutrient supplementation trial (PRECONCEPT): a randomized controlled study. *BMC Public Health*.

[41] Bei-Fan Z. (2002). Cooperative meta-analysis group of the working group on obesity in China. Predictive values of body mass index and waist circumference for risk factors of certain related diseases in Chinese adults–study on optimal cut-off points of body mass index and waist circumference in Chinese adults. *Biomedical and Environmental Sciences*.

[42] Goldstein R. F., Abell S. K., Ranasinha S. (2017). Association of gestational weight gain with maternal and infant outcomes. *Journal of the American Medical Association*.

[43] World Health Organization WHO recommendations on antenatal care for a positive pregnancy experience. http://apps.who.int/iris/bitstream/10665/250796/1/9789241549912-eng.pdf?ua=1.

